# The mutational profile and infiltration pattern of murine MLH1^-/-^ tumors: concurrences, disparities and cell line establishment for functional analysis

**DOI:** 10.18632/oncotarget.10677

**Published:** 2016-07-18

**Authors:** Claudia Maletzki, Franziska Beyrich, Maja Hühns, Ernst Klar, Michael Linnebacher

**Affiliations:** ^1^ Molecular Oncology and Immunotherapy, Department of General Surgery, University of Rostock, 18057 Rostock, Germany; ^2^ Institute of Pathology, University of Rostock, 18057 Rostock, Germany; ^3^ Department of General Surgery, University of Rostock, 18057 Rostock, Germany

**Keywords:** murine tumor models, MMR deficiency, cell line establishment, tumor microenvironment, MSI target genes

## Abstract

Mice lines homozygous negative for one of the four DNA mismatch repair (MMR) genes (*MLH1, MSH2, PMS2, MSH6*) were generated as models for MMR deficient (MMR-D) diseases. Clinically, hereditary forms of MMR-D include Lynch syndrome (characterized by a germline MMR gene defect) and constitutional MMR-D, the biallelic form. MMR-D knockout mice may be representative for both diseases. Here, we aimed at characterizing the MLH1^-/-^ model focusing on tumor-immune microenvironment and identification of coding microsatellite mutations in lymphomas and gastrointestinal tumors (GIT).

All tumors showed microsatellite instability (MSI) in non-coding mononucleotide markers. Mutational profiling of 26 coding loci in MSI^+^ GIT and lymphomas revealed instability in half of the microsatellites, two of them (*Rfc3* and *Rasal2*) shared between both entities. MLH1^-/-^ tumors of both entities displayed a similar phenotype (high CD71, FasL, PD-L1 and CTLA-4 expression). Additional immunofluorescence verified the tumors’ natural immunosuppressive character (marked CD11b/CD200R infiltration). *Vice versa*, CD3^+^ T cells as well as immune checkpoints molecules were detectable, indicative for an active immune microenvironment. For functional analysis, a permanent cell line from an MLH1^-/-^ GIT was established. The newly developed MLH1^-/-^ A7450 cells exhibit stable *in vitro* growth, strong invasive potential and heterogeneous drug response. Moreover, four additional MSI target genes (*Nktr1*, *C8a*, *Taf1b*, and *Lig4*) not recognized in the primary were identified in this cell line.

Summing up, molecular and immunological mechanisms of MLH1^-/-^ driven carcinogenesis correlate well with clinical features of MMR-D. MLH1^-/-^ knockout mice combine characteristics of Lynch syndrome and constitutional MMR-D, making them suitable models for preclinical research aiming at MMR-D related diseases.

## INTRODUCTION

Lawrence Loeb who described possible relationships between DNA replication errors and malignant progression in 1974 initially formulated microsatellite instability (MSI) or mismatch repair deficiency (MMR-D) as a mutator phenotype [1]. Today, much is known about affected proteins and clinical diseases that arise because of MMR-D. Lynch Syndrome (LS, formerly designated as hereditary non-polyposis colorectal cancer) is the most common and MMR-D related cancer-predisposing syndrome. The tumor spectrum is well documented and encomprises multiple colonic and extracolonic (e.g. endometrium and stomach) malignancies [2]. Cancers arise due to a germ-line mutation in one of the MMR genes; to the most part *hMLH1* or *hMSH2* (less frequently *hMSH6* and rarely *hPMS2*) followed by a somatic second hit (chromatin alterations, such as histone modifications or mutations, rarely promoter methylations) that inactivates the relevant MMR gene [3, 4].

As a consequence of MMR-D, unrepaired mutations become scattered throughout the genome, this situation defines the mutator or MSI phenotype and is present in virtually all LS-associated cancers. A unique characteristic of MSI^+^ cancers is the expression of neoantigens due to frameshift mutations (FSM; often 1 or 2 bp deletions) in coding regions of genes. Such FSM constitute true targets for and may contribute to MMR-D-driven mutagenesis and have recently been recognized as ideal (immunological) antigens for vaccination strategies [5].

In recent years, mice lines homozygous negative for each individual DNA MMR protein have been established [6-8]. These models helped in understanding principle biologic processes and pathological consequences which arise because of MMR-D. Here, and similar to the human system in Lynch, *MLH1* and *MSH2* are major players in MMR-D-driven carcinogenesis. Tumors display a high MSI phenotype, with microsatellites being instable in both mono- and dinucleotide repeats [9]. However, very few data exist on the target gene mutational pattern of MMR gene knockout mice. In the only prior study by Woerner et al., FSMs in coding regions of genes were reported [10]. The authors identified MSI target genes in gastrointestinal tumors (GIT) of different MMR-D mouse strains – some of them were even shared between the human and mouse orthologues [10]. These findings are indicative of similar molecular alterations among different species and provide another rationale for knockout mice as models for human MMR-D related diseases. In addition of being representative for LS, these models may be even more akin to a rare inherited human cancer syndrome, in which patients carry biallelic or constitutional MMR-D (CMMR-D) [11, 12]. Very similar to the complex tumor spectrum of affected patients, MMR-D mice predominantly develop early lymphomas followed by GIT at later age. But unlike GIT, the presence of coding FSMs in murine lymphomas has not been investigated yet. It is therefore unknown if both tumor entities have similar mutational profiles. If so, these coding FSMs may act as true (shared) target antigens for functional analysis.

The present study was intended to (I) compare the pattern of coding FSMs in lymphomas and GIT from MLH1^-/-^ mice, (II) examine the interplay between tumor cells and their immune environment and (III) detect more selective target genes in a freshly established MLH1^-/-^ GIT cell line. Identifying concurrences and disparities between both tumor entities helps to gain deeper insights in the mechanisms that underlie MMR-D driven murine carcinogenesis and may pave the way also for preclinical studies on the optimization of MSI-specific vaccination strategies prospectively – for both LS and CMMR-D patients.

## RESULTS

### Tumor spectrum of MLH1^-/-^ mice

**Figure 1:**
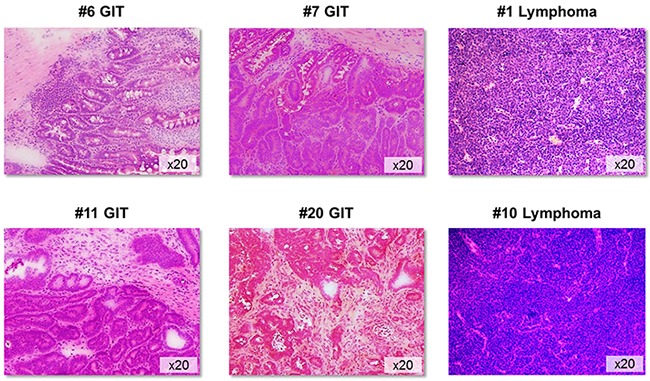
Tumor histology Representative H&E sections of GIT and lymphomas from MLH1^-/-^ mice. GIT appeared as well-differentiated adenocarcinomas showing different invasive potential and morphology. Non-Hodgkin lymphomas were of either B- or T cell origin. Original magnification x20.

For this comparative study, 21gastrointestinal tumors (GIT; from 20 individual mice) and 21 lymphomas (from 11 individual mice) were collected. Lymphomas were detected in the thymus (n=3), spleen (n=7), liver (n=5), kidney (n=3), and duodenum (n=2). Due to the higher aggressiveness of these hematological malignancies, lymphoma development was usually seen before mice were 40 weeks old, while gastrointestinal tumors were only seen at later time (> 42 weeks), which is consistent with the literature [6]. All but one gastrointestinal tumor case were found in the duodenum and histologically defined as well differentiated adenomas (#5) or adenocarcinomas showing different invasive potential and cellular infiltration pattern (Figure 1). The only colorectal cancer (#11) presented with anal bleeding and invasive local tumor growth. Tumors outside the GI tract presented as non-Hodgkin T- or B cell lymphomas (Figure 1).

### MLH1^-/-^ tumor phenotyping & immune cell infiltration

**Figure 2:**
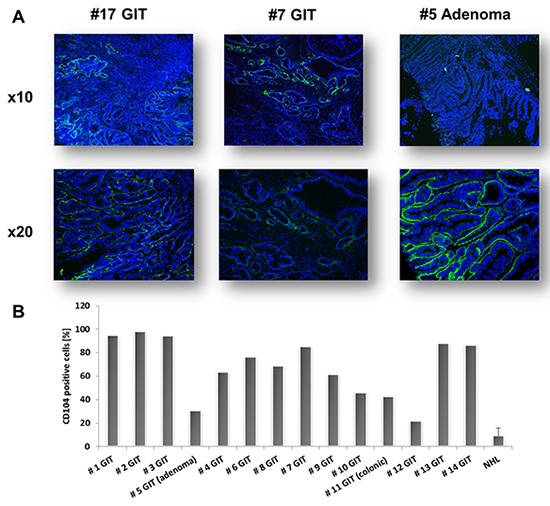
CD104 staining of MLH1^-/-^ GIT **A.** Representative immunofluorescence pictures of CD104-FITC stained GIT. CD104 expression was restricted to epithelial tumor cells. Green – tumor cells; Blue – DAPI **B.** Quantitative analysis of CD104 expression in MLH1^-/-^ GIT as determined by flow cytometry. Note the heterogeneous expression amongst resection specimens, reflecting the different number of tumor cells within the analyzed sample. Negative controls consisted of unstained and/or lymphoma cell stained with CD104.

**Figure 3:**
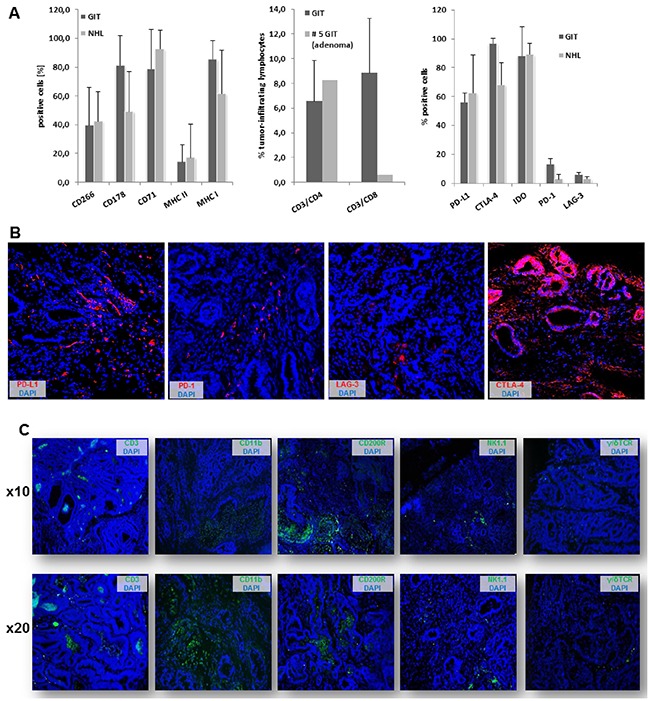
Phenotyping & infiltration pattern **A.** Flow cytometric analysis of GIT and lymphomas showing large phenotypic uniformity between both entities (left chart). Lymphocytic infiltration of primary tumor specimens (middle chart) as well as immune checkpoint expression (right chart) as quantified by flow cytometry (n=5-10 cases/tumor entity). **B.** Immunofluorescence staining of immune checkpoint molecules within GIT specimen. Original magnification x20. **C.** Immune cell infiltration pattern of GIT as determined by immunofluorescence. Original magnification x10 and x20, respectively.

Confirming their epithelial origin, all GIT expressed high amounts of the surface marker CD104, a type I transmembrane glycoprotein (= β4 integrin), that associates with integrin α6 to form the α6/β4 heterodimer. As can be depicted from Figure 2A, CD104 expression was restricted to tumor cells, while no expression was detectable on stromal or infiltrating (immune) cells. As determined by flow cytometry, levels of CD104^+^ tumor cells ranged from 21-98 % (Figure 2B), reflecting the different number of tumor cells within the resection specimen. Additional phenotyping identified high CD178 (FasL) and CD71 expression, the latter being indicative for a high proliferation index [16]. MHC class I expression was also high (about 80%), while only few tumor cells were MHC class II positive. About 40% of the cells expressed the TWEAK receptor CD266 (Figure 3A, left chart). Of note, when analyzing T cell infiltration – a hallmark of human Lynch-associated tumors – considerable levels of both T helper and cytotoxic T cells were found (Figure 3A, middle chart). High tumor-infiltrating T cell numbers were even detectable in a MLH1^+/-^ derived GIT, which was not considered further here (data not shown). Interestingly, the only adenoma (#5) was strongly infiltrated with CD3^+^CD4^+^ T cell, but cytotoxic T cells were virtually absent - partially matching with observations from human Lynch-associated tumors [17].

Comparing these findings with lymphomas, minor differences regarding the surface expression profile are obvious (Figure 3A left chart). NHL where usually CD3^-^CD4^+^CD8^+^ T cell precursors showing intermediate CD168 expression, the receptor for hyaluronan mediated motility (RHAMM). Assessment of immune checkpoint proteins revealed high expression of PD-L1, CTLA-4 (CD152) and IDO in all tumors, while levels of lymphocyte activating gene 3 (LAG-3) and PD-1 were below 20% in both NHL and GIT, with a tendency towards higher expression in the latter (Figure 3A, right chart).

Then, the infiltration pattern of MLH1^-/-^ GI tumors was studied in more detail (Figure 3C). In line with very recent reports on human MSI^+^ CRC, PD-L1 and PD-1 was expressed by stromal cells and infiltrating lymphocytes, while CTLA-4 was highly upregulated on epithelial cells (Figure 3B and [18]). Tumors showed focal CD3^+^ T cell infiltration (CD4^+^ and CD8^+^), usually forming cell clusters. T cells were located at the invasive front and distributed throughout the tumor stroma. Likewise, GIT were infiltrated with CD11b^+^ granulocytes and CD200R^+^ macrophages. Both cell populations have been linked to immune evasion and tumor progression. In line with the natural tumors’ immunosuppressive character, NK cells and γδ T cells were only occasionally detectable within the analyzed resection specimens.

### MSI analysis of murine MLH1^-/-^ tumors

MSI in non-coding regions was assessed by applying a panel of 10 microsatellite markers. Tumors were classified as MSI high if three or more marker were instable.

**Figure 4:**
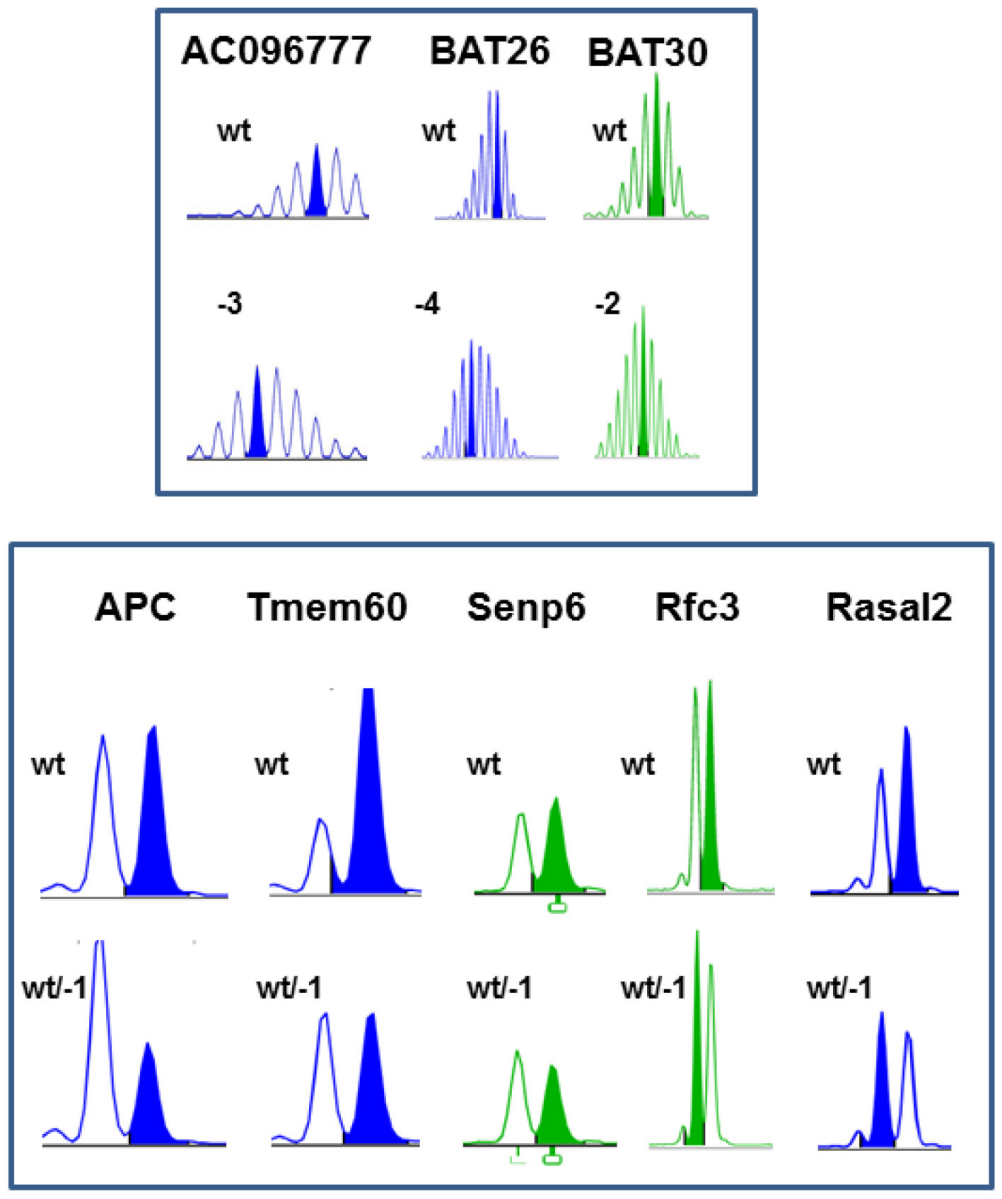
MSI analysis of MLH1^-/-^ tumors Representative pattern of non-coding (upper panel) and coding mononucleotide markers (lower panel). MSI is defined by mono- and/or bialellic band shifts usually presenting as deletions (indicated with minus sign + number) and was determined as described in material & methods.

As anticipated, all GI tumors and lymphomas showed high level of MSI when compared to normal tail DNA, usually occurring as mono- or biallelic deletions (Supplementary Table S3A and S3B). Though only minor variances were found between individual tumor cases, differences concerning the level of instability were obvious, with long repeats being more instable than shorter ones (e.g. Bat30 (A_30_ repeat) and Bat59 (A_59_ repeat) vs. Bat26 (A_26_ repeat) and U12235 (A_24_ repeat), Figure 4, upper panel).

Additional MSI analysis in dinucleotide markers D1Mit79 and D15Mit93 (harboring a CA_31_ and CA_32_ repeat, respectively) revealed some variations between GIT and lymphomas. 35% and 20% of analyzed lymphomas showed instability in at least one dinucleotide marker. Instability in GIT occurred as single base mutation (D1Mit79). The dinucleotide marker D15Mit93 was only instable in one case (#4), while the remaining GIT harbored wildtype D15Mit93.

### Comparative analysis of cMS frameshift mutations in MLH1^-/-^ GIT and NHL

To identify potential target genes of murine MLH1^-/-^ driven tumorigenesis, a panel of mononucleotide markers in coding regions of genes was examined (Supplementary Table S2). Generally, coding frameshift mutations were more frequent in GIT than in NHL (Table 1A and 1B). In GIT, half of the analyzed markers showed instability, present as mono- or biallelic single base deletion (Table 1A and Figure 4, lower panel). Insertions occurred rather infrequently. Of note, each tumor had its own individual mutational profile. Microsatellites of *Phactr4*, *Rfc3*, *Senp6* and *Rasal2* were most affected. The mutation frequency ranged from 40 – 100% for these markers.

**Table 1A:** Frameshift analysis of coding microsatellites in MLH1^-/-^ GI-derived tumors

cMS Marker													
Sample	APC	Tmem60	Senp6	Phactr4	Mdm2	Mdc1	Casc3	SDCCAG1	Rasal2	Tcf7l2	Bend5	NKtr1	Rfc3
**# 1**	wt	wt/-1	wt	-1	wt	wt/-1	wt	wt	wt	wt	wt	wt	wt/-1
**# 2**	wt/-1	wt/-1	wt	wt	wt	wt	wt/-1	wt	wt/-1	wt	wt/-1	wt	wt/-1
**# 3**	wt/-1	wt/-1	wt	-1	wt	wt/-1	wt	wt	wt	wt	wt	wt	wt/-1
**# 4**	wt/-1	wt	-1	wt	wt	wt	wt	wt	wt	wt	wt/-1	wt	wt/-1
# 5*	wt	wt	wt	wt	wt	wt	wt	wt	wt	wt	wt	wt	wt/-1
**# 6**	wt/-1	wt	wt/-1	wt	wt	wt	wt/-1	wt	wt/-1	wt	wt/-1	wt	wt/-1
**# 7**	wt	wt	wt/-1	wt/-1	wt	wt	wt	wt/-1	wt	wt	wt	wt	wt/-1
**# 8**	wt	wt/-1	wt	wt/-1	wt	wt	wt	wt	wt/-1	wt	wt	wt	wt/-1
**# 9**	wt	wt	wt	wt/-1	neg.	wt	wt	wt	wt/-1	wt	wt	wt/-1	wt/-1
**# 10**	wt	wt/-1	wt/-1	wt/-1	wt	wt	wt	wt	wt/-1	wt/-1	wt	wt	wt/-1
**# 11^§^**	wt	wt	wt/-1/-2	wt/-1	wt	wt	wt	wt	wt/-1	wt/-1	wt	wt	wt/-1
**# 11^+^**	wt	wt	wt/-1	wt	del	wt	wt	wt	wt/-1	wt/-1	wt	wt	wt/-1
**# 12**	wt	wt/-1	wt/-1	wt/+1	wt	wt	wt	wt	wt/-1	wt	wt	wt	wt/-1
**# 13**	wt	wt	wt/-1	wt	wt	wt	wt	wt	wt/-1	wt	wt	wt	wt/-1
**# 14**	wt	wt	wt	wt	wt	wt	wt	wt	wt	wt	wt	wt	wt/-1
**# 15**	wt/-1	wt	wt	wt	wt	wt	wt	wt	wt	wt	wt	wt	wt/-1
**# 16**	wt	wt	wt	wt/-1	wt	wt	wt	wt/+1	wt	wt	wt	wt	wt/-1
**# 17**	wt/-1	wt	wt	wt/-1	wt	wt	wt	wt	wt	wt	wt	wt	wt/-1
**# 18**	wt	wt	wt/-1	wt	wt	wt	wt	wt	wt	wt	wt	wt	wt/-1
**# 19**	wt/-1	wt	wt	-1	wt	wt	wt/-1	wt	wt	wt	wt	wt	wt/-1
**# 20**	wt	wt	wt	wt	wt	wt	wt	wt	wt	wt	wt	wt	wt/-1
**frequency [n]**	**7/21**	**6/21**	**9/21**	**11/21**	**2/21**	**2/21**	**3/21**	**2/21**	**9/21**	**3/21**	**3/21**	**1/21**	**21/21**
**frequency [%]**	**33.3**	**28.6**	**42.9**	**52.4**	**9.5**	**9.5**	**14.3**	**9.5**	**42.9**	**14.3**	**14.3**	**4.8**	**100.0**

* – adenoma; § – duodenal; + – colonic; wt – wildtype; - – deletions; + – insertions; neg.- negative, no analyzable signal

**Table 1B:** Frameshift analysis of coding microsatellites in MLH1−/−−derived NHL

cMS Marker													
Sample	APC	Tmem60	Senp6	Phactr4	Mdm2	Mdc1	Casc3	SDCCAG1	Rasal2	Tcf7l2	Bend5	NKtr1	Rfc3
**#1 (duodenal)**	wt	wt/-1	wt	wt	wt	wt	wt	wt	wt/-1	wt	wt	wt	wt/-1
**#2 (duodenal)**	wt	wt	wt	wt	wt	wt	wt	wt	wt	wt	wt	wt	wt/-1
**#3 (thymus)**	wt	wt	wt/-1	wt	wt	wt	wt	wt	wt	wt	wt	wt	wt/-1
**#3 (spleen)**	wt	wt	wt/-1	wt	wt	wt	wt	wt	wt	wt	wt	wt	wt/-1
**#3 (kidney)**	wt	wt	wt/-1	wt	wt	wt	wt	wt	wt/-1	wt	wt	wt	wt/-1
**#3 (liver)**	wt	wt	wt/-1	wt	wt	wt	wt	wt	wt	wt	wt	wt	wt/-1
**#4 (thymus)**	wt/-1	wt	wt/-1	wt	wt	wt	wt/-1	wt	wt	wt	wt	wt	-1
**#4 (spleen)**	wt/-1	wt/-1	wt	wt	wt	wt	wt	wt	wt	wt	wt	wt	-1/-2
**#4 (liver)**	wt/-1	wt/-1	wt	wt	wt	wt	wt/-1	wt/-1	wt	wt	wt	wt	-1
**#5 (spleen)**	wt/-1	wt/-1	wt	wt	wt	wt	wt	wt	wt/-1	wt	wt	wt/-1	wt/-1
**#6 (liver)**	wt	wt/-1	wt	wt	neg.	wt	wt	wt	wt	wt	wt	wt/-1	wt/-1
**#7 (spleen)**	wt	-2	wt	wt	neg.	wt	wt	wt	wt/-1	wt	wt	wt/-1	wt/-1
**#8 (spleen)**	wt	wt	wt	wt	wt	wt	wt	wt/-1	wt	wt	wt	wt	wt/-1
**#9 (kidney)**	wt	wt/-1	wt	wt/+1	wt	wt	wt/-1	wt	wt	wt	wt	wt	wt/-1
**#10 (spleen)**	wt	wt/-1	wt	wt	wt/-1	wt	wt	wt	wt/-1	wt	wt	wt	wt/-1
**#10 (liver)**	wt	wt	wt	wt	wt	wt	wt	wt	wt	wt	wt	wt	wt/-1
**#10 (skin)**	wt	wt/-1	wt	wt	wt/-1	wt	wt	wt	wt/-1	wt	wt	wt	wt/-1
**#11 (thymus)**	wt	wt	wt	wt	wt	wt	wt	wt	wt/-1	wt	wt	wt	wt/-1
**#11 (spleen)**	wt	wt	wt	wt	wt	wt	wt	wt	wt/-1	wt	wt	wt	-1/-2
**#11 (liver)**	wt	wt	wt	wt	wt	wt	wt	wt	wt/-1	wt	wt	wt	-1/-2
**#1 (duodenal)**	wt	wt/-1	wt	wt	wt	wt	wt	wt	wt/-1	wt	wt	wt	wt/-1
**frequency [n]**	**4/21**	**10/21**	**5/21**	**1/21**	**4/21**	**0/21**	**3/21**	**2/21**	**10/21**	**0/21**	**0/21**	**3/21**	**21/21**
**frequency [%]**	**19.0**	**48.0**	**24.0**	**4.8**	**19.0**	**0.0**	**14.3**	**9.5**	**48.0**	**0.0**	**0.0**	**14.3**	**100.0**

wt – wildtype; - – deletions; + – insertions; neg.- negative, no analyzable signal

Among lymphomas, mutations were detected in 10 out of 26 markers tested (Table 1B). Here again, highest frequency was identified in the coding region of the *Rfc3* gene, located on chromosome 5. Hence, all MLH1^-/-^ derived tumors harbored an inactivating mutation in this gene. Functionally, the Rfc3 protein is related to cell cycle and mitosis. Besides, half of the NHL had mutations in *Tmem60* and *Rasal2* microsatellites. The coding repeat of *Tmem60* even shows sequence identity with the human orthologue. Therefore, target genes might at least be partially shared between the different tumor entities and - more importantly - be conserved across species.

### MLH1^-/-^ cell line establishment & characterization

**Figure 5:**
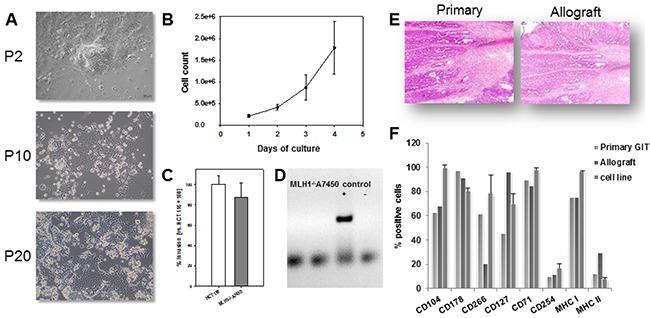
MLH1^-/-^ A7450 cell line characterization **A.** Light microscopy of freshly established tumor cell line (P2) and upon serial passaging (P10 and 20, respectively). The cell line was established from a murine allograft as described in material and methods. Original magnification ×100. **B.** Growth kinetics of cells, counted every 24 hours for four consecutive days using a Neubauer chamber. **C.** Cellular invasiveness was examined using a Matrigel®-based Boyden chamber assay. Quantification of cellular invasiveness was estimated by MTT assay. Data are expressed as percentage invasion versus HCT116 cells (= internal positive control). **D.** Qualitative control as determined by endpoint PCR for exclusion of contaminating mycoplasma **E, F.** Morphology and phenotype of primary GIT in comparison to the allograft. Principal morphological features are retained in the allograft. Comparative phenotyping of the primary, the allograft and the allograft-derived cell line was conducted by flow cytometry using fluorochrom-labeled mAbs as given on the x-axis. (B, D and F) Results show the mean + standard deviation of three independent experiments.

In a first attempt, parts of GI tumor specimens were directly processed for *in vitro* cell line establishment (n=10 individual tumors). This, however, did not yield stable *in vitro* outgrowth. Therefore, parts of the tumor were allografted into immunocompromised mice prior to *in vitro* culture. With this method, allografting and subsequent cell line establishment was successful in 1/10 cases. The cell line MLH1^-/-^A7450 originated from a well-differentiated GI tumor (#4 from our biobank). Initially, cell growth was dependent on coating plates with Matrigel®. At later passages (about P7), cells grew Matrigel®-independently. Determining morphology revealed tight adherence to the bottom of the cell culture flasks. MLH1^-/-^A7450 cells were characterized as epithelial-like cells without contaminating fibroblasts. In the early passages (≤10) multiple morphologically different cell clones were visible (Figure 5A, upper picture). Following serial passaging, they changed their morphology and appeared as rather undifferentiated small, polygonal and round cells not strictly growing in monolayer (Figure 5A). They had a rapid growth kinetic with doubling times of about 24 hours (Figure 5B). Quite in line, the invasive potential was comparable with those of the highly invasive human CRC line HCT116 (87.7% vs. control; Figure 5C). As determined by PCR, the cell line was found free of contaminating mycoplasma (Figure 5D).

### Radiosensitivity and drug response

Upon exposure to increasing doses of X-ray and subsequent *in vitro* culture for 7 days, the MLH1^-/-^A7450 cells began to show impaired adherence. Microscopically, classical signs of radiation induced cell-death were evident, like plasma membrane blebbing, nuclear fragmentation and atypical shapes. LD_50_ value was 12.5Gy and thus slightly higher than for the MMR-proficient control cells (LD_50_ CT26 and Colon-26 cells: 7Gy and 8Gy, respectively).

Next, response towards a panel of clinically approved drugs was examined (Supplementary Table S4) and again compared to response of the control cell lines CT26 and Colon-26. MLH1^-/-^A7450 cells were sensitive towards Gemcitabine, the standard drug for therapy of pancreatic carcinomas. A comparable response was seen upon treatment with Oxaliplatin and Irinotecan. By contrast, cells were largely resistant against 5-FU and Pemetrexed, which is in line with observations on human MMR-D cells [14]. Additive effects were, however, seen in combination therapy, i.e. FOLFOX and FOLFIRI. Of note, all three cell lines showed good response towards the multi-kinase inhibitor Sorafenib, matching with recent reports on Sorafenib-induced killing of human CRC cells [19]. As anticipated, MLH1^-/-^ A7450 cells were highly resistant against the alkylating agents N-Nitroso-N-ethylurea and O^6^-Benzylguanine, while CT26 and Colon-26 cells responded well towards both agents (Supplementary Table S4).

### Comparative analysis: primary GIT vs. allograft and allograft-derived cell line

Morphology of the allograft was compared with the parental tumor (Figure 5E). By means of HE histology, principal architectural and cytological features of the primaries were preserved within the allograft, at least over few passages (≤P4). The epithelial origin was confirmed by positive immunoreactivity for CD104 (>90%; Figure 5F). Further characterization revealed high expression of CD71, CD178 and MHC class I, whereas the TWEAK receptor was differentially expressed between primary and allograft (61.9% vs. 20%). Despite this discrepancy, there was a high phenotypic similarity amongst the parental tumor, the allograft and the corresponding cell line (Figure 5F).

Further comparative characterization included MSI analysis of non-coding and coding FSM. As anticipated, MSI in long mononucleotide repeats increased during passage (primary vs. cell line). Notably, samples showed instability in all markers examined here (Table 2A). Coding FSM analysis yielded different results, with some mutations being only detectable in the allograft and/or the corresponding cell line (Table 2B). Accordingly, prior analysis of the parental tumor might have been confounded by normal epithelial stromal and immune cells. The detected mutations here were found in microsatellites of *Nktr1*, *C8a*, *Taf1b* and *Lig4*. All of them were present as-1bp deletions and mostly affected one allele. FSMs in coding microsatellites of *Rasal2* and *Supt16* were only visible in the allograft but not in the corresponding cell line. By contrast, shifts in fragment lengths of *Tcf7l2* and *Ptpn21* were exclusively seen after *in vitro* culture. Hence, mutations may be present in a single mutated clone that had not been recognized in the resection specimen but gave rise to *in vitro* growth and *vice versa*.

**Table 2A:** Mutational profile of non-coding mono- and dinucleotide repeats in MLH1-/- tumors. Comparison between primary GIT, allograft and allograft-derived cell line

ncMS Marker										
Sample	Bat30	Bat59	Bat26	Bat24	AA003063	U12235	L24372	AC096777	D1Mit79	D15Mit93
**primary**	-1	-3	-1	-1	-1	wt	-3	-6	-1	-2
**allograft**	-4	MSI	-5	-1/-4	-4	-4	-3	-6	-1	-2
**cell line**	-5	-16	-8	-5	-5	-8	-6	-9	wt	-2

wt – wildtype; MSI – microsatellite instable

**Table 2B:** Mutational profile of coding mono- and dinucleotide repeats in candidate target genes of MLH1-/- tumors. Comparison between primary GIT, allograft and allograft-derived cell line

cMS Marker													
Sample	APC	Tmem60	Senp6	Rasal2	Tcf7l2	Bend5	NKtr1	Rfc3	Supt16	C8a	Taf1b	Lig4	Ptpn21
**primary**	wt/-1	wt	-1	wt	wt	wt/-1	wt	wt/-1	wt	wt	wt	wt	wt
**allograft**	wt/-1	wt	wt/-1	wt/-1	wt	wt/-1/-2	wt/-1	wt/-1	-1	MSI	wt/-1	wt/-1	wt
**cell line**	wt/-1	wt	wt/-1	wt	wt/-1	wt/-1	-1	wt/-1	wt	MSI	-1	wt/-1	wt/-1

wt – wildtype; MSI – microsatellite instable

## DISCUSSION

Although the murine MLH1^-/-^ mouse model is well-documented in the literature, very few functional data are available [6, 7]. A systematic analysis of (I) the immunological microenvironment and (II) the cMS frameshift mutational profile has not been performed. Accordingly, the relevance of the immune system for MLH1^-/-^-driven murine tumorigenesis is not well understood. The present study was conducted to comprehensively address this question.

The tumor spectrum observed here closely matched initial descriptions [6]. MLH1^-/-^ mice developed gastrointestinal tumors as well as lymphomas at the same frequency. Although the latter is a rare event in human LS-driven MSI^+^ carcinogenesis, the tumor spectrum and time of onset parallels with patients suffering from biallelic MLH1 mutations [20]. Additionally, principal MMR tumor suppression functions are conserved between men and mice [21]. MLH1^-/-^ mice are thus good models for MMR-D related diseases in general – and maybe even equivalent models to their human constitutional counterpart. With the low overall number of patients and the complex tumor spectrum it is not surprising that the biology of CMMR-D-associated human tumors is not yet understood. To date, tumor-host interactions in CMMR-D patients have not been studied in detail [11, 12]. Initial phenotyping experiments on MLH1^-/-^ tumors revealed surprisingly few differences in surface molecule expression between both tumor entities. In line with their aggressive *in vivo* growth kinetics, the transferrin receptor CD71 and FasL were highly expressed. FasL-expressing tumors are known to establish an immune-privileged status *in situ* to protect themselves from the immune response [22]. By contrast, human MSI^+^ tumor cells do not seem to use FasL to “counterattack” and kill Fas-expressing infiltrating lymphocytes [23]. Additional immunofluorescence analyses confirmed the natural immunosuppressive character of MLH1^-/-^ derived GITs. Tumors were highly infiltrated with CD11b^+^ granulocytes and CD200R^+^ tumor-associated (M2) macrophages. Both cell populations have been linked to immune evasion and tumor progression [24]. *Vice versa* and similar to the situation of human LS, MLH1^-/-^ derived GITs were infiltrated with CD3^+^ T cells, indicating that the immune system may play an active role in tumor surveillance. T cells could even be found within cancer cell nests (clusters), enabling direct contact between tumor and effector cells. Additionally, immune checkpoint molecules were detected within MLH1^-/-^ tumor specimen, which is consistent with findings on human LS and CMMR-D. In humans, high expression of PD-L1 and CTLA-4 as well as good response towards PD-L1 blockade was only recently shown [18, 25 and an overview is given in Table 3].

**Table 3:** Similarities and disparities between mice and men suffering from MLH1 inactivation and accordingly MMR-D

MLH1 loss and functional consequences				
characteristic		Lynch Syndrome	CMMR-D	homozygous knockout mouse model
**clinicopathological characteristics**	**frequency of the underlying mutation among MMR defects**	>40%	~20%	-
	**mean age of onset**	42.4 yrs [37]	7.5 yrs [42]	3.8 months (lymphoma), 8.0 months (GIT) [20 and own observations]
	**tumor incidence**	high, >80%	high, 100%	high, >80%
	**tumor spectrum**	diverse (large and small bowel, endometrium, stomach, kidney, brain)	LS-associated tumors > hematological malignancies> brain tumors	LS-associated tumors ≥ hematological malignancies > others (skin)
	**metastastic spread**	infrequently, syn-or metachronous tumorigenesis is more frequent [26, 37]	yes, very frequent in various organs [42, 43]	not in GIT, only described for lymphomas
	**MHC class I expression**	lost in 30-40% primarily due to β2-microglobulin mutations [35]	unknown	**100% positive**
	**MSI in mononucleotide repeats**	high [27]	divergent results dependent on tumor location and marker panel used to determine MSI (low > high)	high
	**MSI in coding regions of genes**	high, several driver mutations are described (e.g. TGFBR2, AIM2, HT001 and ACVR2A, [27])	largely unknown, one report on TGFβR2 mutations in a PMS2^-/-^ case [44]	some candidate target genes described (*Rfc3*, *Senp6*, *Phactr4*, only GIT, [10]), **novel: Tmem60 and Rasal2 (GIT and lymphoma), *Nktr1*, *C8a*, *Taf1b*, and *Lig4* (only GIT)**
	**karyotype**	near-diploid with few, if any, karyotypic abnormalities [14, 26]	unknown	**near-diploid with few, if any, karyotypic abnormalities (as determined by flow cytometric ploidy analysis)**
**drug response and radiosensitivity**	**common cytostatics**	conflicting results, from high response towards 5-FU > complete resistance, but: good response to oxaliplatin and irinotecan [14, 39]	less known, drug-specific response (GIT and brain tumors seems worse, partial response, if any, while hematological malignancies showed principally good response [11])	**resistance against 5-FU, good response towards Oxaliplatin and Irinotecan**
	**methylating/ alkylating agents**	resistant [39]	resistant (especially O6-methylating agents, like temozolomide [43])	**resistant against SN1 and O6-meythlating agents**
	**radiation**	conflicting results, from no response to good or even better response compared to MMR-proficient tumors (primarily indicated for MMR-D associated rectal cancers (= 8% of all MLH1^-/-^ associated tumors) [40]	radioresistance shown for most of the brain tumors (some with partial response), CRCs do also not seem to respond (partial remission with short recurrence), no large studies on hematological malignancies [11]	**less sensitive than MMR-proficient cells, but not completely resistant**
**tumor microenvironment**	**cytotoxic T- cell infiltration**	high, most of them are activated, primarily clustering at the tumor invasive front [38]	unknown	**high, infiltration increases from adenoma > carcinoma**
	**T helper cell infiltration**	moderate-high, usually co-localizing with CD8^+^ T cells and/or antigen-presenting cells [38]	unknown	**detectable in varying degree, but, if present, primarily clustering at the tumor invasive front**
	**NK cell infiltration**	high, especially in MHC I negative tumors, supposed to be involved in controlling metastasis	unknown	**low > absent**
	**expression of immune checkpoint proteins**	highly upregulated (PD-1, CTLA-4, IDO, LAG-3 in TIL, stroma and invasive front compartments [18, 41])	undescribed, but supposed to be upregulated due to high mutational load and response to PD-L1 blockade [44]	**high expression of CTLA-4 and IDO on tumor cells, upregulated PD-L1 and PD-1 expression in stroma and TIL, but low expression of LAG-3**

TIL - tumor-infiltrating lymphocytes; GIT - gastrointestinal tumor; novel findings are highlighted in bold

The high immunogenicity of human MSI^+^ tumors results from an abundant expression of frameshift neopeptides that are generated as a consequence of insertion/deletion mutations at coding regions of genes [26, 27]. T cells infiltrating these tumors recognize MSI^+^-specific neoantigens – some of them are known to be shared by the majority of MSI^+^ CRCs [28]. The density of tumor-infiltrating lymphocytes even positively correlates with the number of frameshift mutations in MSI^+^ tumors, making them perfect candidate antigens [28]. Consequently, two clinical vaccine trials have just been initiated [trial number: NCT01461148 & NCT01885702]. Results are, however, still pending.

By contrast, little is known about MSI target genes in murine driven tumorigenesis. In a pioneer work by Woerner and colleagues, frameshift mutations in coding regions of six genes from different MMR-D tumors (MLH1^-/-^, MSH2^-/-^, MSH2^loxP/loxP^) were described [10]. Two out of six genes (*Rfc3, Elavl3*) were even conserved in type and length in their human orthologues, suggesting at least partial overlap of MSI target genes and involvement of common oncogenic mechanisms between these two species. In MLH1^-/-^-derived GITs, a high mutation frequency in the microsatellite sequences of *Phactr4* (A_10_), *Senp6* (A_11_) and *Rfc3* (A_10_) was evident [10]. Here, we report several novel findings. First, instability in coding regions of genes is common in both MLH1^-/-^ derived tumor entities analyzed here (i.e. GIT and NHL). We even identified another candidate antigen showing sequence identity with the human orthologue. The protein function of *Tmem60* is largely unknown, but seems to be involved in creatinine production and secretion. It was here found to be mutated in 30% (GIT) and 48% (NHL) of tumors, respectively. Of note and similarly to the surface marker expression analysis, there were no clear differences in mutation frequencies of particular target genes between GITs and lymphomas. An organ-specific mutational profile, as has been proposed for human MSI^+^ tumors in some studies [29], is thus rather unlikely. Interestingly, this finding matches with our previous results on human MMR-D lymphoma/leukemia and CRC cell lines, describing an almost identical mutational susceptibility of specific target genes between these two tumor entities [30]. Second, the most frequently mutated gene was *Rfc3*, supporting the idea that alterations in this gene are important for the development of MLH1^-/-^ tumors – irrespective of organ manifestation. This hypothesis is further corroborated by the presence of *Rfc3* gene mutations already in the adenomas of MLH1^-/-^ mice (case #5 and Table 1A). Third, we identified another potential candidate gene of MLH1^-/-^ driven tumorigenesis, *Rasal2* (*RAS Protein Activator Like 2*). Approximately half of the analyzed GITs and lymphomas harbored an inactivating FSM in an A_8_ repeat of this gene. *Rasal2* encodes a protein that contains the GAP-related domain, a characteristic domain of GTPase-activating proteins. As such, RasGAPs (Ras GTPase-activating proteins) function as tumor suppressors [31]. Indeed, another RasGAP encoding gene, the *NF1* (neurofibromatosis type 1), is a frequent somatic target for CMMR-D-related cancers and it was also shown to be mutated in murine MLH1^-/-^ embryonic fibroblasts [32, 33]. It is thus reasonable to assume that RasGAPs have a causal role in MMR-D driven tumorigenesis. Prospective functional *in vitro* and *in vivo* studies will show if these genes are in fact true target antigens. If so, they are perfect candidates for vaccination strategies.

In principle, an ideal vaccine counteracts tumor heterogeneity and overcomes selection of antigen-negative clones escaping peptide-specific immune responses [34]. Hence, to fully cover a broad tumor antigen profile, additional candidate structures must be identified. Since non-malignant cells and clonal variations might compromise detection sensitivity in analyses of primary tumor material, cell cultures are a perfect starting point for screening of mutational target genes. We therefore established a stable outgrowing MLH1^-/-^ GIT cell line. Although multiple human MMR-D cell lines are available it is noteworthy that this is, to the best of our knowledge, the first murine MMR-D cell line to be described. We would like to state that (I) murine cells can be used for comparative analysis with human material, e.g. for examining the changes in mutational load upon *in vitro* culture, for drug response screening prior to *in vivo* testing and/or detecting acquired resistance mechanisms, (II) they represent a virtually unlimited source of tumor material devoid of non-malignant cells (fibroblasts, infiltrating leukocytes and cells of the blood vessels) for identification of additional coding microsatellites affected by MSI, (III) they can be used as target cells for functional (immunological) assays and (IV) will finally be used as standardized source of MMR-D induced mutations and neoantigens. Basic characterization of the established epithelial cell line revealed a rapid growth kinetic, a heterogeneous response towards standard cancer drugs and large resistance against alkylating agents, which concedes with our findings on human MSI^+^ CRC lines [14 and data not shown].

Are MMR-D mice good models for functional analysis, especially in the context of immune-based therapeutic approaches? Of course, there are species-specific differences in terms of life span, exposure to mutagens and diet that contribute to dissimilarities in cancer formation as for example similar frequencies of lymphomas and GITs in mice vs. rare lymphomagenesis in man, especially in the context of LS (an overview on the most common and novel findings is provided in Table 3). Besides, underlying immunological mechanisms may vary (Table 3). MLH1^-/-^ tumors show high MHC class I expression, while in human LS-associated cancers, β2-microglobulin mutations and accordingly HLA class I loss or downregulation is frequent (up to 40%); most likely resulting from an active immune selection process [35]. One may speculate that tumors in mice with constitutional MMR-D evade immunosurveillance using different mechanisms (as PD-L1 and CTLA-4 expression). Also, murine NK cells are known to be very effective in tumor cell killing. Hence, down regulation or even loss of MHC class I would simply not allow tumors to grow in this microenvironment. Therefore, MHC class I expression is preserved. The observed lymphocytic tumor infiltration pattern (of both CD4^+^ and CD8^+^ cells), the elevated expression of immune checkpoints (particularly PD-L1 and CTLA-4) and the identified FSMs in coding regions of potential MSI target genes supports these ideas; thus being indicative for an active involvement of the immune system on MLH1^-/-^ driven carcinogenesis. This makes them reliable preclinical models for cellular immunotherapies, an especially tempting goal for MMR-D related diseases [5, 30, 36, 38]. Finally, transferring these observations into a mouse model with organ specific MLH1 knockdown will help providing deeper insights into similarities and disparities between individual tumors that arise as a consequence of MMR-D.

## MATERIALS AND METHODS

### Mice breeding & genotyping

Homozygous mice were generated by breeding heterozygous males and females of the ≥F5 generation, hence all mice used in this study were >90% C57BL/6 [6]. All animals received standard laboratory chow and free access to water. Mice breeding took place in the animal facilities (University of Rostock) under specified pathogen-free conditions. Trials were performed in accordance with the German legislation on protection of animals and the Guide for the Care and Use of Laboratory Animals (Institute of Laboratory Animal Resources, National Research Council; NIH Guide, vol.25, no.28, 1996; approval number: LALLF M-V/TSD/7221.3-1.1-053/12). Mlh1 genotyping was done according to [6]. In each generation, offspring of all three classes in the expected ratios were obtained (i.e. about 20-25% homozygous mice).

### Sample collection, murine tumor biobanking, allografting & cell line establishment

Mice were sacrificed before theybecame moribund. After sacrifice, blood samples and tumors were resected. In case of lymphomagenesis, tumors were evident in thymus, spleen, liver, and/or kidney. For detection of GIT, the entire gastrointestinal tract (duodenum, caecum, and colon) was removed and examined for tumor presence under a dissecting microscope. Tumors manifested in both duodenum and colon, with predominance in the former. Upon removal, tumor samples were cut into small pieces and processed further. Parts of the tumor were snap frozen or fixed in Tissue Tek® for molecular and histological analysis. Another piece was further processed for flow cytometric phenotyping. Remaining tumor tissues were frozen viable (FCS, 10% DMSO) at -80°C for subsequent allografting into NMRI Foxn1^nu^ mice as described before [13]. Growing allografts (≥1.500 mm^3^) were resected and processed further for cell line establishment as per description in [14]. In brief, cell culture of the MLH1^-/-^ allograft 7450 (hereafter termed MLH1^-/-^A7450: MLH1^-/-^ = MLH1 knockout; A = allograft + serial breeding number) was started from single cell suspensions, seeded on Matrigel®-coated plates in DMEM medium (+ 10% FCS, 2mM L-glutamine, and antibiotics) and incubated at 37°C in a humidified atmosphere of 5% C0_2_. Medium was changed regularly. Continually growing cell cultures were further passaged and regularly stocked in low passages. Authentication was done by MLH1 genotyping as stated above (verification of complete MLH1 knockout) and flow cytometric phenotyping (e.g. CD104, CD71 and MHC I and Figure 5). Quality control included exclusion of cross-contaminating human cells or mycoplasma by PCR, which was performed every 10^th^ passage [14]. So far, stable outgrowing cultures could be passaged >40 times with no changes in morphology. In some experiments, murine CT26 and Colon-26 cells were used as MMR-proficient controls.

### Microsatellite and coding microsatellite (cMS) frameshift mutation analysis

For detecting MSI in non-coding regions, a panel of microsatellite markers consisting of mononucleotide (n=8) and dinucleotide repeats (n=2) was applied (primer sequences can be taken from Supplementary Table S1) [8, 9]. PCR conditions were: 94°C, 4 min (1 cycle); 94°C, 30 s, 58°C, 45 s and 72°C, 30 s (35 cycles); and 72°C, 6 min (1 cycle). Fluorescently labeled DNA fragments were analyzed on a 3500 Genetic Analyzer. In each reaction, normal tail DNA served as microsatellite stable (MSS) controls. Tumor samples were scored as MSI^+^ if novel peaks were obtained compared to MSS controls or if the ratio of peak areas in MLH1^-/-^ samples and stable controls (tail gDNA of the same mouse) revealed values ≤0.5 or ≥2 [according to 10]. Tumors were scored as MSI^+^ if ≥ 3/10 markers showed instability. To identify potential MLH1 target genes, a panel (n=26) was screened. Genes included in this study were either already described as being candidates [10, 15] or based on extensive database research (own unpublished data). Primers were designed using Primer3 software to yield short amplicons (≤ 200 bp). Primer sequences are listed in Supplementary Table S2. PCR conditions and analysis of results were as described above.

### Flow cytometry on blood & tumor samples

Blood samples were taken routinely from the retrobulbar venous plexus of MLH1^-/-^ mice and stained with a panel of conjugated monoclonal antibodies (mAb) followed by lysis of erythrocytes (FACS Lysing Solution, BD Pharmingen). Negative controls consisted of lymphocytes stained with the appropriate isotypes (BD Pharmingen). For phenotyping of tumor samples, 5 x 10^5^ cells were washed and stained with respective FITC- PE- APC- or PE/Cy-labeled mAbs. Cells were washed and resuspended in 200 μl PBS. Negative controls were stained with the appropriate isotypes. Additionally, cultured tumor cells were phenotyped using a panel of Abs (for details see Figure 5). Cells were analyzed by multicolor flow cytometry on a FACSCalibur Cytometer (BD Pharmingen). Data analysis was performed using CellQuest software (BD Pharmingen).

### Histology & immunofluorescence

Cryostat sections of 4 μm were air-dried and fixed in cold pure methanol for 8 min. Unspecific binding sites were blocked in 2% BSA (2h) followed by incubation with 1 μg of the following FITC-labeled mAbs: CD3ε, CD4, CD11b, γδ TCR, NK1.1 (Immunotools), CD200R, CD104, (Biolegend). Additional stainings included PE-labeled PD-L1, PD-1, LAG-3 and CTLA-4 mAbs (each 1μg, Biolegend). Sections were washed and nuclei were stained with DAPI (0.5 μg/ml). Finally, the target protein was visualized on a fluorescence microscope (Zeiss).

### *In vitro* growth kinetics and matrigel® invasion assay

Population doubling times were determined by viable cells seeded into replicate 25 cm^2^ flasks (0.5 x 10^6^ cells each) and daily counted for four consecutive days. Cellular invasiveness was examined using a Matrigel®-based boyden chamber assay as described [13]. In brief, cells on the lower surface were quantified after 72 hours of incubation by MTT assay and absorbance measurement at 492 nm (reference 620 nm). Data are expressed as percentage invasion versus the highly invasive human colorectal cancer (CRC) line HCT116 [14] set to be = 100 %.

### *In vitro* chemo- and radiosensitivity

Cells were seeded into 96-well microtiter plates (5 x 10^4^ cells/well) and allowed to adhere overnight. Thereafter, triplicate wells were exposed to increasing drug concentrations (pharmacy of the university hospital Rostock) for 72 hours, followed by a second treatment (another 72 hours). Additionally, cells were exposed to two cycles of alkylating agents N-Nitroso-N-ethylurea and O^6^-Benzylguanine. Following these two treatment cycles, medium was removed; plates were carefully washed and stained with crystal violet (0.2%, 10 min). Finally, drug effects from triplicate wells were determined at the level of 50% inhibition (IC_50_) in comparison to control, measured at 570 nm (reference wavelength: 620 nm). Additionally, radiosensitivity was determined upon γ-radiation (ranging from 0 – 100 Gy) and subsequent *in vitro* culture (7 days). LD_50_ (dose of radiation expected to cause death to 50% of all cells) was determined by crystal violet as described.

## SUPPLEMENTARY MATERIALS TABLES



## Abbreviations

CMMR-D: constitutional mismatch repair deficiency
CRC: colorectal cancer
FSM: frameshift mutation
GIT: gastrointestinal tumor
LS: Lynch Syndrome
MMR-D: mismatch repair deficiency
NHL: non-Hodgkin Lymphoma
MSI: microsatellite instability
