# Supplementary material for: The mutational profile and infiltration pattern of murine MLH1-/- tumors: concurrences, disparities and cell line establishment for functional analysis
Source: Oncotarget. 2016 Jul 18;7(33):53583–98. doi: 10.18632/oncotarget.10677 (PMC5288207; doi:10.18632/oncotarget.10677)
Supplement: Supplementary file 1 [file oncotarget-07-53583-s001.pdf]

The mutational profile and infiltration pattern of murine MLH1$^{-/-}$ tumors – concurrences, disparities and cell line establishment for functional analysis

SUPPLEMENTARY TABLES

Supplementary Table S1: Mono- and dinucleotide repeats for detecting MSI in murine GIT and NHL.

Name	Repeat	Product Size [bp]	Primer [5'>3']
AC096777	T_{27}	138	F: 6-Fam-TACAGAGGATTGTCCTCTTGGAG<br>R: GCTGCTTCACTTGGACATTGGCT
L24372	A_{27}	90	F: TAMRA-GGGAAGACTGCTTAGGGAAGA<br>R: ATTTGGCTTTCAAGCATCCATA
Bat24	A_{24}	98	F: 6-Fam-CATAGACCCAGTGCTCATCTTCGT<br>R: CATTCGGTGGAAAGCTCTGA
Bat26	A_{26}	134	F: 6-Fam-TCACCATCCATTGCACAGTT<br>R: CTGCGAGAAGGTACTCACCC
Bat30	A_{30}	82	F: Hex-ATTTGGCTTTCAAGCATCCATA<br>R: GGGAAGACTGCTTAGGGAAGA
Bat59	A_{59}	189	F: Hex-GTAATCCCTTTATTCCATTTAGCA<br>R: GGCTCACAACCATCCGTAACAAGA
AA003063	A_{23}	87	F: 6-FAM-ACGTCAAAAATCAATGTTAGG<br>R: CAGCAAGGGTCCCTGTCTTA
U12235	A_{24}	86	F: Hex-GCTCATCTTCGTTCCCTGTC<br>R: CATTCGGTGGAAAGCTCTGA
D1Mit79	CA_{31}	150	F: TAMRA-GACCTGGAAGTTGGAAACCA<br>R: TGATCCAGAACCTCTGCCTT
D15Mit93	CA_{32}	144	F: TAMRA-AAGAATTGGGGTGGGTAAGG<br>R: CATGTGCAGAATACTTACACATATGC

Supplementary Table S2: Coding mononucleotide repeats for detecting MSI in murine MLH1$^{-/-}$ GIT and lymphomas and function of the resulting protein.

Name	Repeat	Product Size [bp]	Primer [5'>3']	Protein function
APC	A_8	189	F: FAM-ATTTCTCTGGATCGCCTTT<br>R: GCGAATCTTTCCCGTAATCA	*Adenomatous Polyposis Coli* tumor suppressor, keeping cells from growing and dividing too fast or in an uncontrolled way
Tmem60 **sequence conserved in human and mouse**	A_9	164	F: FAM-CTTTGACCCTCGACATGGAT<br>R: CAGCCAGTAAGGCCCATAAA	*Transmembrane Protein 60* suspected to affect creatinine production and secretion

(*Continued*)

Name	Repeat	Product Size [bp]	Primer [5’>3’]	Protein function
Senp6	A_{11}	177	F: HEX-GCTGTGACTGATTCGAGTGC<br>R: GCCATTCTCACTTTGGGTTC	*SUMO1/Sentrin Specific Peptidase 6*<br>Protease, structurally related to ubiquitin
Phactr4	A_{10}	185	F: TAMRA-ATCTTCAAGCCGTGGAAATG<br>R: TTTAATGCAGCAGGCTCAG	*Phosphatase And Actin Regulator 4*<br>Interacts with actin and protein phosphatase 1, indirectly controlling cell cycle progression
Mdm2	A_{8}	198	F: TAMRA-CGCAGGACAAAGACGAGAGT<br>R: TCATTTGGATTGGCTGTCTG	*Proto-Oncogene, E3 Ubiquitin Protein Ligase*<br>promotes tumor formation by targeting tumor suppressor proteins, such as p53, for proteasomal degradation
Mdc1	C_{8}	188	F: HEX-GAAAGGCTGGTTGCTTCTTG<br>R: ACTGATGATCTCGCCCATCT	*Mediator Of DNA-Damage Checkpoint 1*<br>required to activate the intra-S phase and G2/M phase cell cycle checkpoints in response to DNA damage
Casc3[15]	C_{8}	140	F: TAMRA-TGTCACAC TTCCATAGAAACACACC<br>R: GAGGACTCCCCAGCCGGT	*Cancer Susceptibility Candidate 3*<br>core component of the exon junction complex deposited, functions in nonsense-mediated mRNA decay
SDCCAG1[15]	A_{11}	132	F: FAM-GCATAGGCTGACAGGCTGAGAT<br>R: TGCCAGCATTGAGAACAGTGAT	*Serologically Defined Colon Cancer Antigen 1*<br>component of the ribosome quality control complex
Rasal2[15]	A_{8}	151	F: FAM-CAAACTGGCGACCAGTCACAC<br>R: TAGCCTTCCACCTCTTCATAGCA	*RAS protein activator like 2*<br>encodes a RasGAP-like protein containing the GAP-related domain
Tcf7l2[15]	T_{8}	119	F: HEX-CACCTTGTATGTAGCGAACGCA<br>R: TCAACTTGCCGTGAGCATTAGA	*Transcription Factor 7 like 2*<br>high mobility group (HMG) box-containing transcription factor implicated in blood glucose homeostasis
Bend5	A_{8}	113	F: FAM-CTGGGGAACAGATGTTCTGA<br>R: CTCTGACGATGCTGAGCTTG	*BEN domain containing 5*<br>largely unknown
Nktr1	A_{9}	95	F: HEX-GCGAGACACTCTGATGGTCA<br>R: TGTCTTCTGCAATGTTTCTGC	*Natural Killer Cell Triggering Receptor*<br>membrane-anchored protein with a hydrophobic amino terminal domain and a cyclophilin-like PPIase domain; Component of a putative tumor-recognition complex
Rfc3[10]	A_{10}	165	F: HEX-TTGGCAGTAGTGATTTGGTGA<br>R: AGGAGCAGTTTACCTGGGATT	*Replication Factor C (Activator 1) 3*<br>Binds PCNA (proliferating cell nuclear antigen) to increase the stability of DNA polymerases
Supt16	A_{8}	85	F: FAM-TGAGAAAGTTCGGCATAGCA<br>R: AGAAGGATCTGCCCCAGCTA	*no information available*

(*Continued*)

Name	Repeat	Product Size [bp]	Primer [5'>3']	Protein function
C8a	T_8	100	F: TAMRA-CCTGGCGGATACTTCAATCT<br>R: ACACCAGCTGAGTGGGACTT	*Complement Component 8, Alpha Polypeptide*<br>C8 participates in the formation of the membrane attack complex
Taf1b	A_8	180	F: FAM-CATGTGACAACTTACGAA GAATCA<br>R: TCCTTTCTTGGATCCTGAGC'	*TATA Box Binding Protein (TBP)-Associated Factor B, RNA Polymerase I*<br>Component of RNA polymerase I core factor complex, plays a key role in multiple steps during transcription initiation
Lig4	A_9	133	F: FAM-TGAAGCCAAGGTGCTTACAG<br>R: TGGGTTATTAACTGAAGAAGGCT	*DNA Ligase IV*<br>DNA repair enzyme, joins single-strand breaks in a double-stranded polydeoxynucleotide in an ATP-dependent reaction
Ptpn21[10]	A_8	176	F: FAM-TTTGAAAGGTGTCTGGGTACG<br>R: CGCTCTGGTGGACACTTCTT	*protein tyrosine phosphatase, non-receptor type 21*<br>signaling molecule regulating a variety of cellular processes including cell growth, differentiation, mitotic cycle, and oncogenic transformation

Supplementary Table S3A: Frameshift analysis of non-coding microsatellites in MLH1$^{-/-}$ GI-derived tumors.

	ncMS									
**Sample**	**Bat30**	**Bat59**	**Bat26**	**Bat24**	**AA003063**	**U12235**	**L24372**	**AC096777**	**D1Mit79**	**D15Mit93**
**# 1**	-1	-1	-1	wt	wt	wt	wt	-1	wt	wt
**# 2**	-1	-1	-1	-1	-1	wt/-1	-1	-1	wt	wt
**# 3**	-1	wt	wt	wt	-1	wt/-1	wt	wt	wt	wt
**# 4**	-1	-3	-1	-1	-1	wt	-1	-2	-1	-2
**# 5***	-1	-2/-7	wt	wt	wt	wt/-1	-2	wt	wt	wt
**# 6**	-1	-3	-1	wt/-1	-1	-1	-1	-1	wt	wt
**# 7**	-2/-4	-2/-10	-1/-5	-2	-1/-3	-2	-1/-5	-2	wt	wt
**# 8**	-2	-2/-9	-1	-1	-1	wt	-2	-2	-1	wt
**# 9**	-1	-1/-3	-1	-1	-1	wt	-1	-1	wt	wt
**# 10**	-2	-8	-2	-2	-2	-2	-3	-3	-1	wt
**# 11§**	-1	-7	-1	-1	-1	wt	-2	-1	wt	wt
**# 11+**	-2	-9	-3	-1	-1	wt	-2	-3	wt	wt
**# 12**	-3	-8	-2	-2	del	del	del	-5	wt	wt
**# 13**	wt	-5	-2	-1/-3	-1	-2	-1	-3	+1	wt
**# 14**	-3	MSI	-2/-7	MSI	-2	MSI	-5	-5	wt	wt
**# 15**	-2	MSI	-2	-1	-2	-2	-2	-1/-5	-1	wt
**# 16**	-6	MSI	-4	-1/-6	-2/-5	MSI	-1/-7	-5	wt	wt
**# 17**	-2	MSI	-1	-1	-1	MSI	-2	-2	wt	wt
**# 18**	wt	MSI	wt	-1	-1	MSI	-1	-1	-1	wt
**# 19**	-3	MSI	-3	-1	-1	MSI	-3	-2	-1	wt
**# 20**	-1	-2	-2	-1	-1	wt/-1	-1	-3	-1	wt
**frequency [n]**	**20/21**	**20/21**	**18/21**	**18/21**	**19/21**	**15/21**	**19/21**	**19/21**	**8/21**	**1/21**
**frequency [%]**	**95.24**	**95.24**	**85.71**	**85.71**	**90.48**	**71.43**	**90.48**	**90.48**	**38.10**	**4.76**

* – adenoma; § – duodenal; + – colonic; wt – wildtype; - – deletions; + – insertions; MSI – microsatellite instable (not quantitatively analyzable)

Supplementary Table S3B: Frameshift analysis of non-coding microsatellites in MLH1$^{-/-}$ derived NHL.

	ncMS									
**Sample**	**Bat30**	**Bat59**	**Bat26**	**Bat24**	**AA003063**	**U12235**	**L24372**	**AC096777**	**D1Mit79**	**D15Mit93**
**#1 (duodenal)**	-2	-5	-1	-3	wt	wt	-1	-1	wt	wt
**#2 (duodenal)**	wt	-4	-1	wt/-8	-1	wt	wt/-7	wt/-6	-1	wt
**#3 (thymus)**	-2	-3	-1	-1	-1	-1	-1	-2	wt	wt
**#3 (spleen)**	-1	-2	-1	-1	-1	-1	-1	-1	wt	wt
**#3 (kidney)**	-1	-2	-3	-1	-1	wt/-1	-1	-1	wt	wt
**#3 (liver)**	-1	-2	-1	-2	-1	1	-1	-1	wt	wt
**#4 (thymus)**	-2	-8	-2	-2	-1	-2	-2	-3	wt	wt/+2
**#4 (spleen)**	-1	-8	-1	-2	-1	+1	-2	-2	wt	wt/+2
**#4 (liver)**	-1	-8	-1	-2	MSI	MSI	MSI	-6	wt	+2
**#5 (spleen)**	-1	-5	-1	-2	-2	-2	-2	-2	-5	+2
**#6 (liver)**	-2	-5	-2	-1	-1/-2	-1	-3	-4	wt	wt
**#7 (spleen)**	-2	-5	-2	-1	-2	-1	-3	-4	wt	wt
**#8 (spleen)**	-2	-12	-1	-3	-2	wt	-2	-3	wt	wt
**#9 (kidney)**	-3	-14	-3	-2	-1	-3	-3	-1	-1	wt
**#10 (spleen)**	-2	-7	-3	wt	-1	wt	-3	-2	+1	wt
**#10 (liver)**	-2	-7	-3	wt	-1	wt	-3	-2	+1	wt
**#10 (skin)**	-1	-6	-3	wt	del	wt	del	del	+1	wt
**#11 (thymus)**	-3	-7	-2	-2	-1	-3	-3	-1	wt	wt
**#11 (spleen)**	-2	-6	-2	-2	-1	-3	-3	-1	wt	wt
**#11 (liver)**	-2	-5	-3	-2	-1	-3	-3	-1	wt/-2	wt
**frequency [n]**	**19/20**	**20/20**	**20/20**	**17/20**	**19/20**	**14/20**	**20/20**	**20/20**	**7/20**	**4/20**
**frequency [%]**	**95.00**	**100.00**	**100.00**	**85.00**	**95.00**	**70.00**	**100.00**	**100.00**	**35.00**	**20.00**

wt – wildtype; - – deletions; + – insertions; MSI – microsatellite instable (not quantitatively analyzable)

Supplementary Table S4: IC_{50} values of antitumor drugs evaluated for MLH1$^{-/-}$ A7450 cells in comparison with MMR-proficient control cells.

drug	IC_{50} value		
	MLH1$^{-/-}$A7450	CT26	Colon-26
5-FU	280 ng/mL	10 ng/mL	80 ng/mL
Gemcitabine	0.015 µg/mL	1 µg/mL	1.2 µg/mL
Oxaliplatin	0.8 µg/mL	4 µg/mL	4 µg/mL
Cisplatin	2 µg/mL	2.8 µg/mL	0.25 µg/mL
Irinotecan	32 µM	100 µM	35 µM
Pemetrexed	100 µM	0.1 µM	32 nM
Sorafenib	7.5 µM	2.5 µM	<1 µM
FOLFOX	additive effects*	synergistic effects*	additive effects*
FOLFIRI	additive effects*	additive effects*	additive effects*
N-Nitroso-N-ethylurea	>500 µM	10 µM	10 µM
O^6-Benzylguanine	>250 µM	5 µM	8.9 µM

*compared to monotherapy
